# Supplementary material for: Permafrost in the Cretaceous supergreenhouse
Source: Nat Commun. 2022 Dec 26;13:7946. doi: 10.1038/s41467-022-35676-6 (PMC9792593; doi:10.1038/s41467-022-35676-6)
Supplement: Supplementary file 1 — Supplementary Information [file 41467_2022_35676_MOESM1_ESM.pdf]

Supplementary Information

Permafrost in the Cretaceous supergreenhouse

Juan Pedro Rodríguez-López[1,2,3], Chihua Wu[1,4*], Tatiana A. Vishnivetskaya[5], Julian B. Murton[3], Wenqiang Tang[1,6], Chao Ma[4]

[1]PAGODA Research Group (Plateau & Global Desert Basins Research Group), Institute of Sedimentary Geology, Chengdu University of Technology, Chengdu 610059, China.

[2]Department of Geology, Faculty of Science and Technology, University of the Basque Country (UPV/EHU), Ap. 644, E-48080 Bilbao, Spain.

[3]Permafrost Laboratory, Department of Geography, University of Sussex, Brighton BN1 9QJ, UK.

[4]State Key Laboratory of Oil and Gas Reservoir Geology and Exploitation, Institute of Sedimentary Geology, Chengdu University of Technology, Chengdu 610059, China

[5]Center for Environmental Biotechnology, University of Tennessee, Knoxville, TN 37996, USA.

[6]Research Institute of Petroleum Exploration and Development, PetroChina Southwest Oil and Gas Field Company, Chengdu, Sichuan 610051, China.

*Corresponding author. Email: wuchi-hua@foxmail.com

Supplementary Note 1-Geological setting.

This study focuses on the Cretaceous Luohe Fm, in the Ordos Basin, North China Craton (NCC) (Supplementary Fig. 1a–d). As one of the oldest continental crustal blocks on Earth, the NCC is thought to have experienced intense lithospheric thinning during the Mesozoic to early Cenozoic (ref.[1] and references therein). After its initial formation in the late Palaeoproterozoic, the NCC maintained stability until the early Mesozoic, and accumulated thick neritic clastic rocks and carbonates[2]. However, due to the late Mesozoic subduction of the Palaeo-Pacific plate and the closure of the Mongolia–Okhotsk Ocean, the dynamic system of the NCC underwent a major transformation, which led to the destruction of the eastern NCC or eastern Plateau and culminated in the early Cretaceous (ca. 125 Ma)[1–3]. Many extensional structures appeared within the NCC, including metamorphic core complexes, detachment faults, and a series of faulted basins (e.g., Ordos Basin), accompanied by magmatic activities and variation in the physical properties of the lithospheric mantle during the early Cretaceous[2].

The NCC is separated from the South China Block (SCB) by the Qinling–Dabie orogenic belt (Supplementary Fig. 1a)[4]. The Ordos Basin (OB) is located in the western NCC and bounded by the Helan–Liupan Mountains to the west, the Lvliang Mountains to the east, the Qinling Mountains to the south, and the Yinshan Mountains to the north. This basin only contains lower Cretaceous Zhidan Group red-bed successions, in ascending order: the Yijun Fm, the Luohe Fm, the Huanhe–Huachi Fm, the Luohandong Fm, and the Jingchuan Fm (Supplementary Fig. 1b)[5]. The Yijun Fm is dominated by red conglomerates with coarse sandstone lenses of alluvial origin. The Luohe Fm, which is the focus of the present study, consists of red sandstone with larger tabular/trough crossbedding deposited in an erg system, whereas the Huanhe–Huachi Fm is characterized by red sandstones and mudstones of a fluvial–lacustrine origin. The Luohandong Fm is dominated by red sandstone and mudstones with larger crossbedding deposited in an erg/fluvial system, and the Jingchuan Fm consists of variegated mudstone with sandstones or yellow conglomerates, indicating a fluvial–lacustrine/alluvial origin. The Zhidan Group contains ichthyolite (*Lycoptera–Sinamia*), conchostracan (*Orthestheria*), Ostracoda (*Lycopterocypris–Cypridea–Rhinocypris*), and sporopollen assemblages (*Cicatricosisiporites–Densoisporites*), all indicating an early Cretaceous age (ref.[5], and references therein). In

addition, palaeomagnetic studies date the Yijun Fm as late Berriasian (ca.142.06–141.48 Ma), the Luohe Fm as late Berriasian–Valanginian (ca.141.48–133.94 Ma), and the Huanhe–Huachi Fm as Hauterivian (ca.133.94–129.73 Ma)[5].

The Lower Cretaceous Luohe sandstone is mainly composed of 430–110 m thick cross-beds generated by migration and climbing of huge sand dunes (up to a maximum height of 352 m)[6,7]. The palaeowind orientations in the Ordos Basin during the Cretaceous were easterly or north-easterly, suggesting that the prevailing winds were westerlies[8] (Supplementary Fig. 2a). The Valanginian–Hauterivian annual mean surface air temperature (at 1.5 m height above the ground surface) for the desert basin has been modelled as ≤0ºC (Supplementary Fig. 2b)[9].

Supplementary Note 2–Stratigraphic architecture and sedimentology of the Luohe Fm.

The Luohe Fm attains thicknesses of >30 m in the studied outcrops and shows superimposed sets of large-scale (gigantic) cross-bedded sandstones with a maximum thickness of 10 m. The sandstones are fine-grained, very well sorted and show a reddish brown colour, minor faults and fractures and a pristine preservation of small-scale to large-scale sedimentary structures (Supplementary Fig. 3).

Superimposed cross-bedded sets show an average thickness of 3 m (range 1–10 m) (Supplementary Fig. 3a). Lamina sets in the foresets show a constant direction of dip and coherent orientation with alternating dm- to cm-thick tabular laminae with cm- to mm-thick laminae; a single lamina can be traced laterally for more than 20 m and shows inverse grading. Geometrical relationships between laminae of underlying and overlying laminasets are variable; dip angles vary from 1º to 30º among superimposed cross-bedded sets. The bottomsets of cross-bedded sets show pinching out of wedge-shaped downward termination of foreset laminae (Supplementary Fig. 3b and c) interbedded with inversely graded superimposed lamina sets forming subcritically climbing translatent strata (Supplementary Fig. 3d).

The outcrops of the Luohe Fm display large-scale cross-bedded sets showing a hierarchy of stratigraphic surfaces. Internally, foresets show inclined surfaces dipping in the same direction as the foreset lamination covered by downlapping lamination of the overlying cross-bedded set (reactivation surfaces 'R' in Supplementary Fig. 3a). Foreset deposits also contain surfaces (superimposition surfaces 'S' in Supplementary Fig. 3a) that cross-cut reactivation surfaces and above which overlying cross-bedded sets show downlapping geometries. Both types of surfaces (R and S) are contained and crosscut by main bounding stratigraphic surfaces (marked 'IS'), which dip in the opposite direction to the dip direction of the large foresets (Supplementary Fig. 3a). The highest order hierarchical surfaces are aeolian supersurfaces ('SS') that crosscut the IS surfaces as well as the sets of cosets that IS bound and contain surfaces R and S (Supplementary Fig. 5).

Sandstone wedges appear always to be associated with SS surfaces in the studied outcrops (Supplementary Figs. 4 and 5). The wedges penetrate the underlying cross-bedded sandstones and are truncated by SS surfaces and overlain by downlapping large-scale cross-bedded facies (Fig. 1b and Supplementary Figs. 4b–d, and 5a–c).

A sedimentary succession 30 m thick formed by fine-grained very well-sorted sandstones showing large-scale (gigantic) cross-bedding is interpreted to have resulted from the accumulation of migrating aeolian dunes[10–13]. The inclined sandy wedges in the foresets indicate grain flow deposits (Supplementary Fig. 3b, and c) merging to wind-ripple laminations, indicative of a common facies pattern observed in aeolian dune bottomsets[14–17] due to alternation of grain flow caused by gravitational instability and wind reworking on aeolian dune foresets[18]. The variable dips among cross-bedded sets correspond to different orientation of three-dimensional sections of the sets, showing low angles when sections are perpendicular to prevailing wind direction and high angles in sections parallel to palaeowind transport direction[19]. Subcritically climbing translatent strata showing inverse grading (Supplementary Fig. 3d) resulted from the migration of wind ripples [20–24].

The hierarchy of bounding surfaces indicates that dunes had crescentic crests and superimposed smaller dunes migrating on a major parent bedform, together conforming to a complex dune (draa)[9,13,14,25,26]. Surfaces "R" are reactivation surfaces formed by reactivations of foreset sedimentation and/or the effect of secondary winds on the dune foreset[14,26,27]. Surfaces "S" are superimposition surfaces due to the migration of superimposed dunes on a parent bedform[12,26]. "IS" surfaces are interdune surfaces, formed by the migration of an interdune depression over the stoss slope of the underlying aeolian dune [25,28,29]. Overall, these were complex dunes, and the interdune surfaces constituted interdraa surfaces[30]. The main bounding surfaces (surface SS) are aeolian supersurfaces that separate genetically related aeolian desert sequences (erg sequences) and are considered the response of allogenic forcing on the desert system[31] water-level changes, including climate change, synsedimentary tectonics and erg migration[31–37].

In summary, the studied outcrops of the Luohe Fm in the Ordos Basin represent the sedimentary record of migrating draas (complex dunes that can attain heights of >350 m in modern deserts worldwide) whose development was interrupted by changing allogenic conditions that triggered the formation of aeolian supersurfaces (SS in Supplementary Fig. 5). The studied sandstone wedges appear always in discrete stratigraphic horizons associated with erg sequence boundaries (aeolian supersurfaces, SS) (Supplementary Figs 4b–d, and 5a–c).

Supplementary Note 3–Sandstone wedge sedimentology.

Sandstone wedges have been identified in three different outcrops of the Luohe Fm. Outcrop one (Fig. 1b, and Supplementary Figs. 4, and 6e) shows two distinct levels of wedges separated by trough-cross bedded aeolian sandstones with tangential downlapping of aeolian toeset sediments on the wedge tops. Outcrop two (Supplementary Figs. 3 and 6a–d) shows two wedges penetrating aeolian dune cross-bedded sets. Outcrop three (Figs. 1c–d, 2 a–e and Supplementary Fig. 5) shows ten wedges concentrated in two discrete horizons bounding three draa successions (Fig. 1c–d). The wedges are up to 2 m high and are estimated to be up to c. 1 m wide (orthogonal to their axial planes). The heights are thought to be minimum values because the tops of all of the wedges are truncated by erosion surfaces, and so an unknown amount of wedge material has been eroded away.

The sandstone wedges shows similar morphological and sedimentological characteristics in the three outcrops (Fig. 2 and Supplementary Fig. 6): (i) stratigraphic occurrence in discrete horizons covered by undeformed sediments (Fig. 1c–d, Supplementary Figs. 5 and 6a, b, and e); (ii) sharp sides with an overall wedge shape that generally tapers downwards (Fig. 2a–e and Supplementary Figs. 5, 6a, b, e); (iii) vertical to subvertical internal lineations and fractures parallel to the wedge sides occur within some wedges (Fig. 2d and Supplementary Fig. 6b–e); (iv) involutions with a U-shaped to gently concave-upward lamination occur in the upper part of some wedge infills (Fig. 2d, and Supplementary Fig. 6b, e) and the top section with infilling discordant and onlapping aeolian sediments (Fig. 2a–e and Supplementary Fig. 6a–e); (v) downward termination of some wedges in sandstone fingers penetrating down into underlying cross-bedded sandstones (Fig. 2a–e and Supplementary Fig. 6a–e); (vi) wedges show evidence of cracking and rotation in both wedge upper margins (Supplementary Figure 6c–e); (vii) intraclasts derived from the wedge upper margins occur within some wedge infills indicate that some host sediment has collapsed into the wedge and rotated (Supplementary Fig. 6); (viii) the overall shapes of many wedges is irregular, with opposite sides often being non-symmetrical and abruptly changing orientation and/or width (Fig. 2a, b and Supplementary Fig. 6e).

We interpret the sandstone wedges as permafrost-related wedges after considering two lines of evidence: (i) the wedges do not show the typical features of giant desiccation cracks, or tectonic/tensional structures; (ii) the wedges show numerous morphological affinities to relict sand wedges and composite-wedge pseudomorphs of Proterozoic to Holocene age, and their palaeogeographic and palaeoclimate setting correlates well with recent permafrost–aeolian plateau desert systems from the western Himalayas. We use the standard terminology for periglacial wedges set out by (ref.[38]).

The morphology and infills of the wedges from the Cretaceous aeolian dune sandstones from the Luohe Fm are rare in the sedimentary record of aeolian dunes worldwide[10]. Desiccation cracking often occurs in desert basins and can generate giant cracks, often polygonal, both in ancient[39] and recent desert basins[40]. However, such cracking develops in basin floors formed by silty and muddy (cohesive) playa lake systems rather than sandy aeolian dune deposits. Ancient faulting and fracturing in aeolian sandstones are normally postdepositional due to both tectonic deformation[41] and weathering[42]. Tension (tensile) cracks can develop due to gravitational slope instability but not in flat, nearly palaeohorizontal interdune surfaces over a non-cohesive substrate (aeolian sands) and lacking synsedimentary infill. The geometries, dimensions, and penetration depths of all these cracking mechanisms are incompatible with the features and morphometric properties of the wedges observed in the Luohe Fm.

Supplementary Note 4–Quaternary analogue of the Cretaceous permafrost wedges.

A Quaternary analogue for the Cretaceous permafrost wedges is provided by sand wedges that penetrate cross-bedded aeolian dune facies from the Late Pleistocene Kittigazuit Fm, Hadwen Island, Tuktoyaktuk Coastlands, NT, Canada (Supplementary Fig. 7)[43]. The aeolian sand-dune deposits of the Kittigazuit Fm are widespread in the Tuktoyaktuk Coastlands[44–46]. The dunes developed in a permafrost environment, and their deposits are frozen, bonded with pore ice, and contain visible ice veins and lenses (1–5 mm thick); thaw depths that can reach 1.5 m or more[44]. The Kittigazuit Fm contains permafrost wedges that crosscut the host cross-bedded aeolian dune facies[47] (Supplementary Fig. 7). The upper part of the wedges is truncated, and the tops of the wedges resumed upward growth within aeolian sand-sheet deposits. Deposition of aeolian sand within the Kittigazuit Fm took place under conditions of continuous permafrost and shows morphological affinities with the permafrost wedges observed in the Cretaceous Luohe Fm of China, including (i) permafrost wedges overlain by laminated aeolian sands (1 in Supplementary Fig. 8a, b); (ii) local upturned strata in aeolian host sands that show a sharp contact with wedge margins (2 in Supplementary Fig. 8a, b); (iii) wedge showing a margin with a stepped shape (3 in Supplementary Fig. 8a, b); (iv) sharp truncation of the wedge's top covered by downlapping laminated aeolian sandstones (4 in Supplementary Fig. 8a, b); (v) rejuvenated top central part of the wedge (5 in Supplementary Fig. 8a, b); (vi) downward narrowing of the wedge with clear internal vertical lamination (6 in Supplementary Fig. 8a, b); (vii) wedges overlain by non-deformed

and laminated aeolian sands (7 in Supplementary Fig. 8a, b); and (viii) local downturned strata of host aeolian sandstone (8 in Supplementary Fig. 8a, b).

Additional similarities between permafrost wedges in the Pleistocene Kittigazuit Fm and the Cretaceous Luohe Fm are shown in Supplementary Fig. 9. They include: (i) sand veins in the wedge toe (1 in Supplementary Fig. 9a, b); (ii) downward bending of wedge toe into laminated aeolian sandstones (2 in Supplementary Fig. 9a, b); (iii) sharp and nearly orthogonal contact between wedge margins and stratification in the host aeolian sandstone (3 in Supplementary Fig. 9a, b); (iv) stepped margin of the wedge entering into the wedge infill (4 in Supplementary Fig. 9a, b); (v) internal vertical lamination parallel to the wedge margins (5 in Supplementary Fig. 9a, b); (vi) downward bending of internal lamination towards the wedge toe (6 in Supplementary Fig. 9 a, b); and (vii) upward widening of the wedge and a stepped margin (7 in Supplementary Fig. 9a, b).

Supplementary Note 5–Recent environmental analogue from the Western Himalayas.

The cold aeolian dunefield suffered two transgressions in 2007 and 2013 expanding the lake shore as recorded in satellite imagery (Fig. 4, and Supplementary Fig. 10). These transgressions partially submerged the aeolian dunefield margin and flooded some interdune depressions. It is possible to observe an ice floe in the lake surface (Fig. 4a–d and ice floes in interdune areas of the adjacent dunefield (Fig. 4e–g).

Supplementary Note 6–Magnetostratigraphy and cyclostratigraphy.

The revised magnetostratigraphic studies were based on the geomagnetic polarity time scale (MHTC12) (ref. [48] and cyclostratigraphic analysis (Supplementary Fig. 11).

Previous systematic palaeomagnetic work on the Early Cretaceous was performed in the Shaozhai area of Ordos Basin[5]. Such work relied on the geomagnetic polarity time scale GTS2004 (ref.[49]), whereas we used MHTC2012 to obtain a more accurate dating by magnetostratigraphy. Based on the virtual geomagnetic polarity (VGP) latitudes (Supplementary Fig. 11), 24 pairs of normal (N1 to N24) and reverse (R1 to R24) magnetic polarities are observed in the Shaozhai Section[5]. The observed magnetic polarities are correlated with chrons CM5n–CM12r.1n of the geomagnetic polarity time scale MHTC12, yielding the age range of 134.0 to 126.1 Ma for the measured section (Supplementary Fig. 11), while the top boundary of the Luohe Fm is 129.4 Ma.

GR logs of Well Lingtai and Well Wuqi were selected for cyclostratigraphic analysis of the early Cretaceous in Ordos Basin (Supplementary Fig. 1d). One of them, Well Lingtai, is adjacent to the Shaozhai Section where previous work on magneticstratigraphy was carried out, while the Well Wuqi is close to the section where the permafrost wedges crop out (Supplementary Fig. 1d).

Analysis of the cyclostratigraphy of the Luohe–Yijun Fm enables the construction of a floating astronomical time scale for the Lower Cretaceous strata in the Ordos Basin of China. We selected logs from two wells (Well Lingtai and Well Wuqi) to investigate their cyclostratigraphy. The obvious 7.54 cm/kyr sedimentation rate (H0 significance levels lower than 0.1%) and apparent 30.49 m sedimentation cycle (confidence levels greater than 95%) in the Well Lingtai (Supplementary Fig. 12a) provide a significant 405-kyr astronomical signal. The filtering of 405-kyr cycle was used to convert depth to time[50], thus establishing a 4.34 myr long floating astronomical time scale for the Well Lingtai. Using the same method to the Luohe Fm of the Well Wuqi created a 4.19 Myr long floating astronomical time scale (sedimentation rate: 8.24 cm/kyr, dominated cycle: 33.09 m) (Supplementary Fig. 12b). The cyclostratigraphic age defined by the top boundary of the Luohe Fm (ca. 129.4 Ma, in MHTC12), combined with the identification of an excellent stratigraphic datum for lithostratigraphic correlation, defined by the stratigraphic contact between purplish red medium bedded, middle-grained sandstones, and the underlying red thick-bedded fine-grained sandstones with large high-angle tabular cross-bedding or medium-bedded conglomerates, allowed us to obtain an astronomically corrected duration of 133.74–129.4 Ma for the Luohe–Yijun Fm in Well Lingtai, and 133.59–129.4 Ma for the Luohe Fm in Well Wuqi, respectively (Fig. 7 and Supplementary Fig. 11). Differences between the age framework established by the two wells and our recalibrated magnetic stratigraphic age is an error of just under 405-kyr, indicating that the results are plausible. In addition, it is noteworthy that a significant obliquity signal can be traced after 133 Ma in the evolutionary FFT spectral analysis of Well Wuqi (Fig. 7).

In geologic history, the climatic significance of the obliquity is that it drove the occurrence of glacial periods, representing a cooling or cold climate[51,52]. The Eocene–Oligocene transition marks the passage from early greenhouse conditions to modern icehouse ones in the Cenozoic, while the significant signal of obliquity in some sedimentary records is an indication of strong astronomical constraints during this climatic transition[53,54]. Similarly, in both the Oligocene–Miocene as well as the Middle Pleistocene, the emergence of the glacial period shows a clear obliquity signal in the astronomical cycle[51,52]. Further, the Late Ordovician Hirnantian glaciation has been confirmed to have been forced by obliquity[55]. Therefore, our study may provide a record of glaciation during the warm Early Cretaceous, and which may be correlated with the Hauterivian cold snap, before the early Barremian pulse[56].

By Gaussian filtering, the 405-kyr cycle exhibits a change from high to low amplitude at the bottom of the Luohe Fm, while the ~40-kyr filter maintains a relatively low amplitude over time. A similar phenomenon has been documented in the case of the Mi-1 glaciation event[51]. Our record then confirms a glacial event, which correlates with global palaeoclimate proxies worldwide (Fig. 7), including IRD and glendonites in Svalbard, Australia and Alaska[57–59], and postdates the Weissert Event[60,61].

A minimum eccentricity will place the Earth in an unusually limited position for seasonal variation. The lower the obliquity, the less solar irradiation the polar regions receive, which favours the creation of ice sheets[51,62]. We presume that from 132.49 Ma onwards, the polar summer continued to cool, and the ice sheets expanded in the Northern Hemisphere with the appearance of minimum eccentricity and a decline in obliquity. After this, the eccentricity increased rapidly and the obliquity changed from low to high amplitude, the orbital–climate effect then gradually disappeared, and the ice volume decreased[51,62]. The evolutionary FFT spectrum and the variation of eccentricity and obliquity cycles can be well correlated with the horizon of the permafrost sandstone wedges, which also confirms the accuracy of the revised palaeomagnetic age framework.

Supplementary Information References

1. Zhu, R. X., Yang, J. H. & Wu, F. Y. Timing of destruction of the North China Craton. *Lithos* **149**, 51–60 (2012).
2. Zhu, R. X., Chen, L., Wu, F. Y. & Liu, J. L. Timing, scale and mechanism of the destruction of the North China Craton. *Sci. China Earth Sci.* **54**, 789–797 (2011).
3. Wu, F. Y., Lin, J. Q., Simon, A. W., Zhang, X. O. & Yang, J. H. Nature and significance of the Early Cretaceous giant igneous event in eastern China. *Earth Planet Sci. Lett.* **233**, 103–119 (2005).
4. Li, J., Zhang, Y., Dong, S. & Johnston, S. T. Cretaceous tectonic evolution of South China: a preliminary synthesis. *Earth-Sci. Rev.* **134**, 98–136 (2014).
5. Huang, Y. B. The origin and evolution of the desert in southern Ordos in early Cretaceous: Constraint from Magnetostratigraphy of Zhidan Group and magnetic susceptibility of its sediment. Doctoral Dissertation. Lanzhou University (2010).
6. Ma, J. Sedimentary basin analysis of the Cretaceous ancient desert in the Ordos Basin. Master's thesis, China University of Geosciences (2020).
7. Xing, L. D. et al. The non-avian theropod track Jialingpus from the Cretaceous of the Ordos Basin, China, with a revision of the type material: implications for ichnotaxonomy and trackmaker morphology. *Palaeoworld* **23**, 187–199 (2014).
8. Hasegawa, H. et al. Drastic shrinking of the Hadley circulation during the mid-Cretaceous Supergreenhouse. *Clim. Past* **8**, 1323–1337 (2012).
9. Lunt, D. J. et al. Palaeogeographic controls on climate and proxy interpretation. *Clim. Past* **12**, 1181–1198 (2016).
10. Rodríguez-López, J. P., Clemmensen, L. B., Lancaster, N., Mountney, N. P. & Veiga, G. D. Archean to Recent aeolian sand systems and their preserved successions: current understanding and way forward. *Sedimentology* **61**, 1487–1534 (2014).
11. Kocurek, G. in *Sedimentary Environments: Processes, Facies and Stratigraphy (*ed Reading, H.G.) 125– 153 (Blackwell, Oxford, 1996).
12. Mountney, N. P. in *Facies Models Revisited, SEPM Mem., 84* (eds Walker, R. G. & Posamentier, H.) 19–83 (Society for Sedimentary Geology, 2006).
13. Mountney, N. P. Periodic accumulation and destruction of aeolian erg sequences in the Permian Cedar Mesa Sandstone, White Canyon, southern Utah, USA. *Sedimentology* **53**, 789–823 (2006).
14. Rodríguez-López, J. P., Meléndez, N., de Boer, P. L. & Soria, A. R. Controls on marine-erg margin cycle variability: aeolian-marine interaction in the Mid- Cretaceous Iberian Desert System, Spain. *Sedimentology* **59**, 466–501 (2012).
15. Rodríguez-López, J. P. et al. Aeolian construction and alluvial dismantling of a fault bounded intracontinental aeolian dune field (Teruel Basin, Spain); a continental perspective on Late Pliocene climate change and variability. *Sedimentology* **59**, 1536–1567 (2012).
16. Wu, Ch. et al. Late Cretaceous climbing erg systems in the western Xinjiang Basin. Palaeoatmosphere dynamics and East Asia margin tectonic forcing on desert expansion and preservation. *Mar. Petrol. Geol.* **93**, 539–552 (2018).
17. Rodríguez-López, J. P. & Wu, Ch. Recurrent deformations of aeolian desert dunes in the cretaceous

of the South China Block: trigger mechanisms variability and implications for aeolian reservoirs. *Mar. Petrol. Geol.* **119**, 104483 (2020).
18. Loope, D. B., Rowe, C. M. & Joeckel, R.M. Annual monsoon rains recorded by Jurassic dunes. *Nature* **412**, 64–66 (2001).
19. Li, G. et al. Mid-Cretaceous aeolian desert systems in the yunlong area of the Lanping Basin, China: implications for palaeoatmosphere dynamics and paleoclimatic change in east Asia. *Sediment. Geol.* **364**, 121–140 (2018).
20. Hunter, R. E. Basic types of stratification in small eolian dunes. *Sedimentology* **24**, 361–387 (1977).
21. Fryberger, S. G., Al-Sari, A. M. & Clisham, T. J. Eolian dune, interdune, sand sheet, and siliciclastic sabkha sediments of an offshore prograding sand sea, Dhahran Area, Saudi Arabia. *Am. Assoc. Petrol. Geol. Bull.* **67**, 280–312 (1983).
22. Clemmensen, L. B. & Abrahamsen, K. Aeolian stratification and facies association in desert sediments, Arran basin (Permian), Scotland. *Sedimentology* **30**, 311–339 (1983).
23. Fryberger, S. G. & Schenk, C. J. Pin stripe lamination: a distinctive feature of modern and ancient eolian sediments. *Sediment. Geol.* **55**, 1–15 (1988).
24. Scherer, C. M. S. et al. Stratigraphy and facies architecture of the fluvial-aeolian-lacustrine Sergi Formation (Upper Jurassic) Recômcavo Basin, Brazil. *Sediment. Geol.* **194**, 169–193 (2007).
25. Brookfield, M. E. The origin of bounding surfaces in ancient aeolian sandstones. *Sedimentology* **24**, 303–332 (1977).
26. Rodríguez-López, J. P., Meléndez, N., de Boer, P. L. & Soria, A. R. Aeolian sand sea development along the mid-Cretaceous western Tethyan margin (Spain): erg sedimentology and palaeoclimate implications. *Sedimentology* **55**, 1253–1292 (2008).
27. Mountney, N. P. & Thompson, D. B. Stratigraphic evolution and preservation of aeolian dune and damp/wet interdune strata: an example from the Triassic Helsby Sandstone Formation, Cheshire Basin, UK. *Sedimentology* **49**, 805–833 (2002).
28. Kocurek, G. Interpretation of ancient eolian sand dunes. *Annu. Rev. Earth Planet. Sci.* **19**, 43–75 (1991).
29. Fryberger, S. G. in *Characterization of fluvial and aeolian reservoirs. Geol. Soc. London Spec. Pap., vol. 73* (eds North, C. P. & Prosser, D. J.) 167–197 (Geological Society, London, London, 1993).
30. Clemmensen, L. B. & Hegner, J. Eolian sequence and erg dynamics: the Permian Corrie Sandstone, Scotland. *J. Sed. Petrol.* **61**, 768–774 (1991).
31. Rodríguez-López, J. P., Meléndez, N., de Boer, P. L., Soria, A. R. & Liesa, C. L. Spatial variability of multicontrolled aeolian supersurfaces in central-erg and marine erg-margin systems. *Aeolian Res.* **11**, 141–154 (2013).
32. Talbot, M. R. Major bounding surfaces in aeolian sandstones – a climatic model. *Sedimentology* **32**, 257–265 (1985).
33. Kocurek, G. First-order and super bounding surfaces in eolian sequences – bounding surfaces revisited. *Sediment. Geol.* **56**, 193–206 (1988).
34. Havholm, K. G. et al. in *Aeolian Sediments. Ancient and Modern. Int. Assoc. Sedimentol. Spec. Publ., 16* (eds Pye K. & Lancaster, N.) 87–108 (Blackwell Scientific Publications, Oxford, 1993).
35. Kocurek, G. & Havholm, K. G. in: *Siliciclastic Sequence Stratigraphy: Recent Developments and Applications. AAPG Mem., 58* (eds Weimer P. & Posamentier, H.W.) 393–409 (The American Association of Petroleum Geologists, Tulsa, 1993).
36. Mountney, N. P. & Howell, A. Aeolian architecture, bedform climbing and preservation space in the Cretaceous Etjo Formation, NW Namibia. *Sedimentology* **47**, 825–849 (2000).
37. Veiga, G. & Spalletti, L. A. The Upper Jurassic (Kimmeridgian) fluvial-aeolian systems of the southern Neuquen Basin, Argentina. *Gondwana Res.* **11**, 286–302 (2007).
38. Murton, J. B. in: Encyclopedia of Quaternary Science. Vol. 3 (eds Elias, S. A. & Mock, C. J.) 436–451 (Elsevier, Amsterdam, 2013).
39. Loope, D. B. & Haverland, Z. E. Giant desiccation fissures filled with calcareous eolian sand, Hermosa Formation (Pennsylvanian), southeastern Utah. *Sediment. Geol.*, **56**, 403–413 (1988).
40. El Maary, M., Pommerol, A. & Thomas, N. Analysis of polygonal cracking patterns in chloride-bearing terrains on Mars: Indicators of ancient playa settings. *J. Geophys. Res. Planets* **118**, 2263–2278 (2013).
41. Sternlof, K. R., Rudnicki, J. W. & Pollard, D. D. Anticrack inclusion model for compaction bands in sandstone. *J. Geoph. Res.* **110**, B11403 (2005).

42. Chan, M. A., Yonkee, W. A., Netoff, D. I., Seiler, W. M. & Ford, R. L. Polygonal cracks in bedrock on Earth and Mars: Implications for weathering. *Icarus* **194**, 65–71 (2008).
43. Murton, J. B., Bateman, M. D., Waller, R. I. & Whiteman, C. A. in: *GeoQuebec 2015. GeoQuebec 2015: 7th Canadian Permafrost Conference, 20–23 September, 2015, Quebec City, Canada.* (Quebec City, Canada, 2015).
44. Dallimore, S. R., Wolfe, S. A., Matthews, J. V. Jr. & Vincent, J-S. Mid-Wisconsinan eolian deposits of the Kittigazuit Formation, Tuktoyaktuk Coastlands, Northwest Territories. Canada. *Can. J. Earth Sci.* **34**, 1421–1441 (1997).
45. Murton, J. B., Frechen, M. & Maddy, D. Luminescence dating of Mid- to Late Wisconsinan aeolian sand as a constraint on the last advance of the Laurentide Ice Sheet across the Tuktoyaktuk Coastlands, western Arctic Canada. *Can. J. Earth Sci.* **44**, 857–869 (2007).
46. Murton, J. B. Stratigraphy and Palaeoenvironments of Richards Island and the Eastern Beaufort Continental Shelf during the Last Glacial-Interglacial Cycle. *Permafr. Periglac. Process.* **20**, 107–125 (2009).
47. Murton, J. B. & Bateman, M. D. Syngenetic sand veins and anti-syngenetic sand wedges, Tuktoyaktuk Coastlands, western Arctic Canada. *Permafr. Periglac. Process.* **18**, 33–47 (2007).
48. Malinverno, A., Hildebrandt, J., Tominaga, M. & Channell, J. E. T. M-sequence geomagnetic polarity time scale (MHTC12) that steadies global spreading rates and incorporates astrochronology constraints. *J. Geophys. Res.* **117**, B06104 (2012).
49. Gradstein, F. M., Ogg., J. G. & Smith. A. G. *A Geologic Time Scale 2004* (The Cambridge University Press, Cambridge, 2004).
50. Hinnov, L. A., Hilgen, F. J. in: *The Geologic Time Scale 2012* (eds Gradstein, F.M., Ogg, J. G. & Schmitz, M. & Ogg, G. M.) 63–83 (Elsevier, Amsterdam, 2012).
51. Zachos, J. C., Shackleton, N. J., Revenaugh, J. S., Pälike, H. & Flower, B. P. Climate Response to Orbital Forcing Across the Oligocene-Miocene Boundary. *Science* **292**, 274–278 (2001).
52. Drysdale, R. N. et al. Evidence for obliquity forcing of glacial termination II. *Science* **325**, 1527–1531 (2009).
53. Abels, H. A., Dupont-Nivet, G., Xiao, G., Bosboom, R. & Krijgsman, W. Step-wise change of Asian interior climate preceding the Eocene–Oligocene Transition (EOT). *Palaeogeogr. Palaeoclimatol. Palaeoecol.* **299**, 399–412 (2011).
54. Ao, H. et al. Orbital climate variability on the northeastern Tibetan Plateau across the Eocene–Oligocene transition. *Nat. Commun.* **11**, 1–11 (2020).
55. Zhong, Y., Wu, H., Fan, J., Fang, Q. & Cao, L. Late Ordovician obliquity-forced glacio-eustasy recorded in the Yangtze block, south China. *Palaeogeogr. Palaeoclimatol. Palaeoecol*. **540**, 109520 (2019).
56. Malkoč, M. & Mutterlose, J. The early Barremian warm pulse and the late Barremian cooling: a high-resolution geochemical record of the Boreal Realm. *Palaios* **25**, 14–23 (2010).
57. Alley, N. F., Hore, S. B. & Frakes, L. A. Glaciations at high-latitude Southern Australia during the Early Cretaceous. *Aust. J. Earth Sci.* **67**, 1045–1095 (2020).
58. Vickers, M. L. et al. The duration and magnitude of Cretaceous cold events: Evidence from the northern high latitudes. *Geol. Soc. Am. Bull.* **131**, 1979–1994 (2019).
59. Keller, M. A. & Macquaker, J. H. S. in: *Studies by the U.S. Geological Survey in Alaska: US Geological Survey Professional Paper 1814-B, v. 15* (ed Dumoulin, J. A.) 1–35 (US Geological Survey, US Department of The Interior, Reston, 2015).
60. Cavalheiro, L. et al. Impact of global cooling on Early Cretaceous high *p*CO2 world during the Weissert Event. *Nat. Commun.* **12**, 5411 (2021).
61. Erba, E., Bartolini, A. & Larson, R. L. Valanginian Weissert oceanic anoxic event. *Geology* **32**, 149–152 (2004).
62. Li, M. et al. Obliquity-forced climate during the early Triassic hothouse in China. *Geology* **44**, 623–626 (2016).
63. Xi, D. P., Wan, X. Q., Li, G. B. & Li, G. Cretaceous integrative stratigraphy and timescale of China. *Sci. China Earth Sci.* **62**, 112–134 (2019).

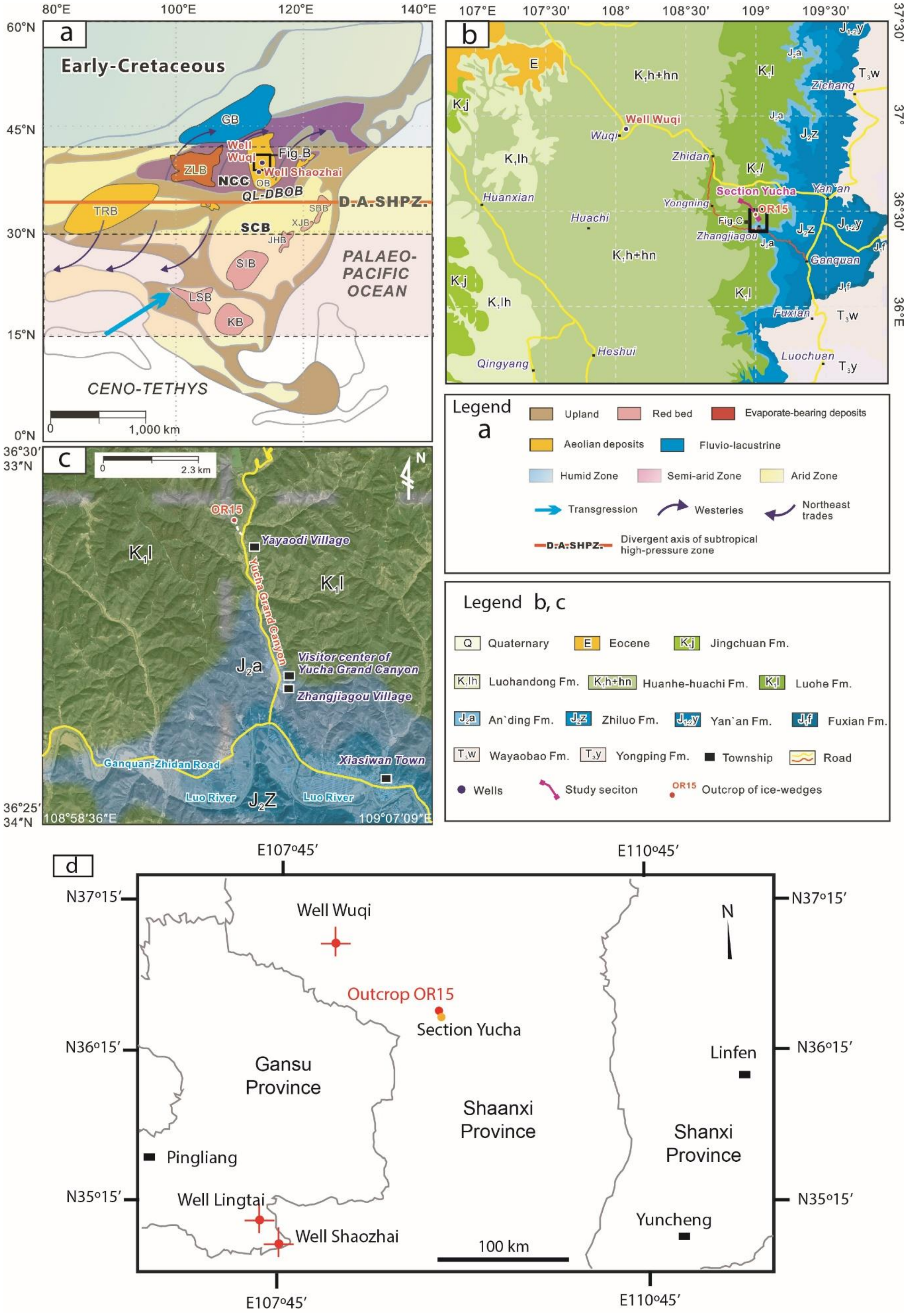

a
Early-Cretaceous
GB
Well Wuqi
Fig.B
ZLB
Well Shaozhai
NCC
OB
QL-DBOB
D.A.SHPZ.
TRB
SBB
XJB
SCB
JHB
SIB
PALAEO-PACIFIC OCEAN
LSB
KB
CENO-TETHYS
1,000 km
b
Well Wuqi
Wuqi
Zhidan
Huanxian
Huachi
Yongning
Section Yucha
OR15
Fig.C
Zhangjiagou
Yan'an
Zichang
Ganquan
Fuxian
Luochuan
Heshui
Qingyang
c
2.3 km
OR15
Yayaodi Village
Yucha Grand Canyon
Visitor center of Yucha Grand Canyon
Zhangjiagou Village
Ganquan-Zhidan Road
Xiasiwan Town
Luo River
Legend a
Upland
Red bed
Evaporate-bearing deposits
Aeolian deposits
Fluvio-lacustrine
Humid Zone
Semi-arid Zone
Arid Zone
Transgression
Westeries
Northeast trades
Divergent axis of subtropical high-pressure zone
Legend b, c
Quaternary
Eocene
Jingchuan Fm.
Luohandong Fm.
Huanhe-huachi Fm.
Luohe Fm.
An`ding Fm.
Zhiluo Fm.
Yan`an Fm.
Fuxian Fm.
Wayaobao Fm.
Yongping Fm.
Township
Road
Wells
Study seciton
Outcrop of ice-wedges
d
Well Wuqi
Outcrop OR15
Section Yucha
Gansu Province
Shaanxi Province
Shanxi Province
Linfen
Pingliang
Well Lingtai
Well Shaozhai
Yuncheng
100 km

Supplementary Fig. 1 | Geological and geographic setting of the Asian interior during the Early Cretaceous. a, Palaeogeographic reconstructions, spatio–temporal distribution of climate-indicative sediments and palaeowind directions (modified after refs.[8,16]). **b,** Geological sketch map of the Ordos Basin. **c,** Location of the wedge structures outcrops in the Ordos Basin. OB: Ordos Basin; ZLB: Zoulang Basin; GB: Gobi Basin; TRB: Tarim Basin; LSB: Lanping–Simao Basin; SIB: Sichuan Basin; KB: Korat Basin; JHB: Jianghan Bain; XJB: Xingjiang Basin; SBB: Subei Basin; NCC: North China Craton; SCB: South China Block; QL-DBOB: Qinling–Dabie Orogenic Belt. **d,** Geographic location of the studied outcrops and wells.

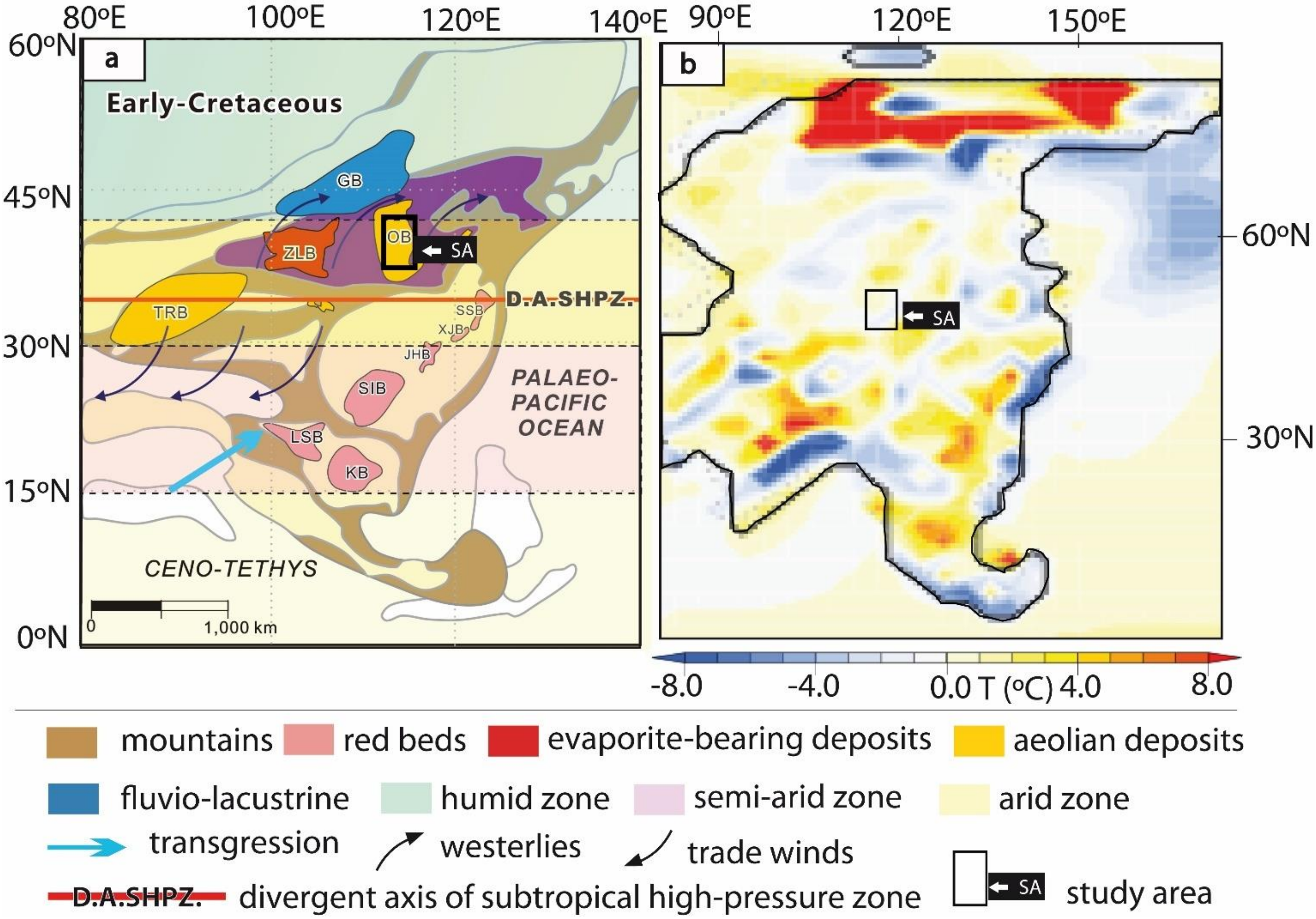

Supplementary Fig. 2 | Palaeoclimate and palaeogeograhy of the Asian interior during the Early Cretaceous. a, Palaeogeographic reconstructions, spatio–temporal distribution of climate-indicative sediments and palaeowind directions[16]. **B,** SA: study area. Annual mean surface air temperature (at 1.5 m above the ground surface) for the Valanginian–Hauterivian stages (adapted after ref.[9]). Under CC Attribution 3.0 License. https://creativecommons.org/licenses/by/3.0/

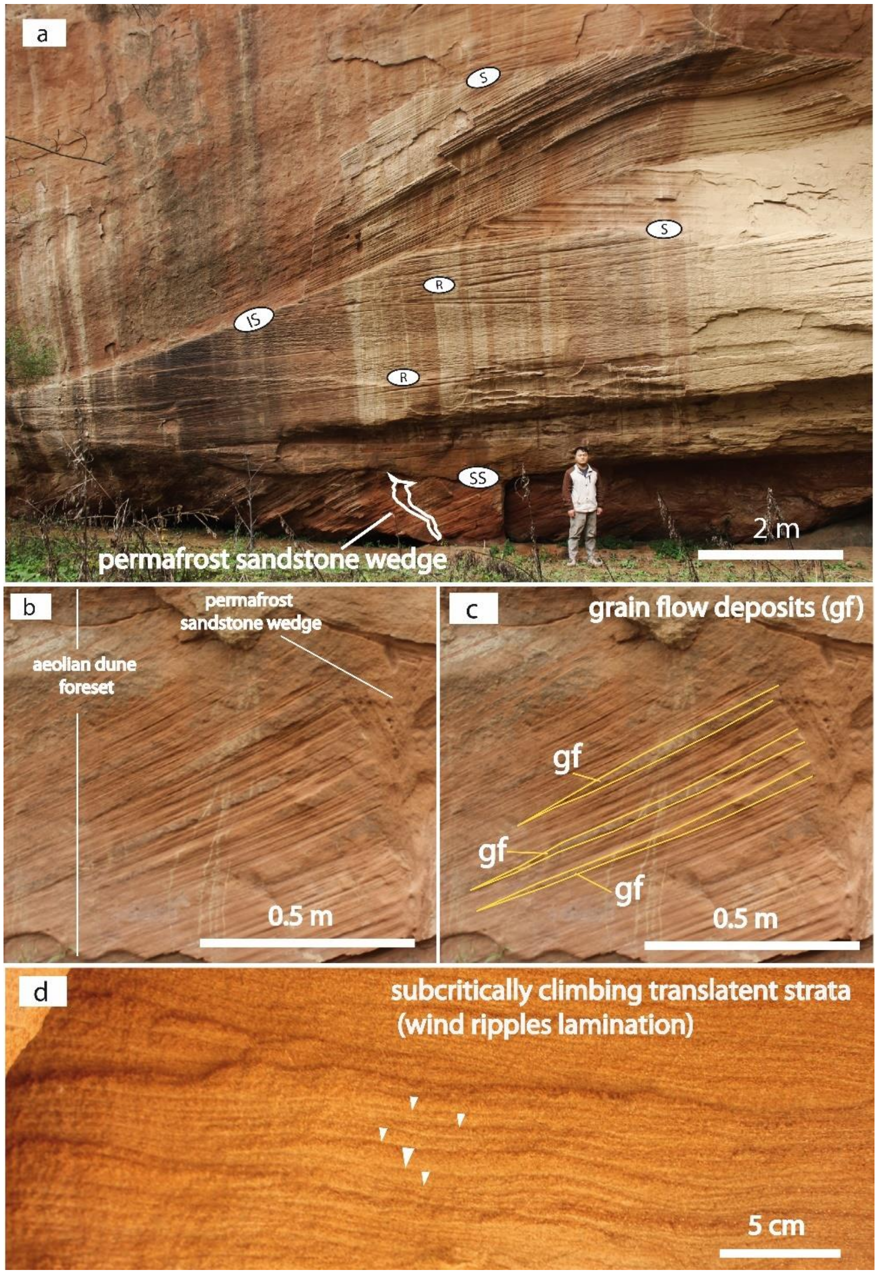

Supplementary Fig. 3 | Aeolian facies of the Luohe Fm. a, Aeolian stratigraphic architecture of complex aeolian dunes (draas) in the Luohe Fm. Sandstone wedge marked in white truncated along its top by an aeolian supersurface (SS). "R" refers to reactivation surfaces, and "S" to superimposition aeolian surfaces. "IS" marks the position of an interdune (interdraa) surface. **b,** Close-up view from (**a**) showing the stratigraphic relationship between the wedge and the host aeolian dune foreset facies. **c,** Grain flow wedges (gf). **d,** detail of wind ripple lamination showing inverse grading (inverted white triangles mark the coarsening upwards trend of every lamina) and forming subcritically climbing translatent strata.

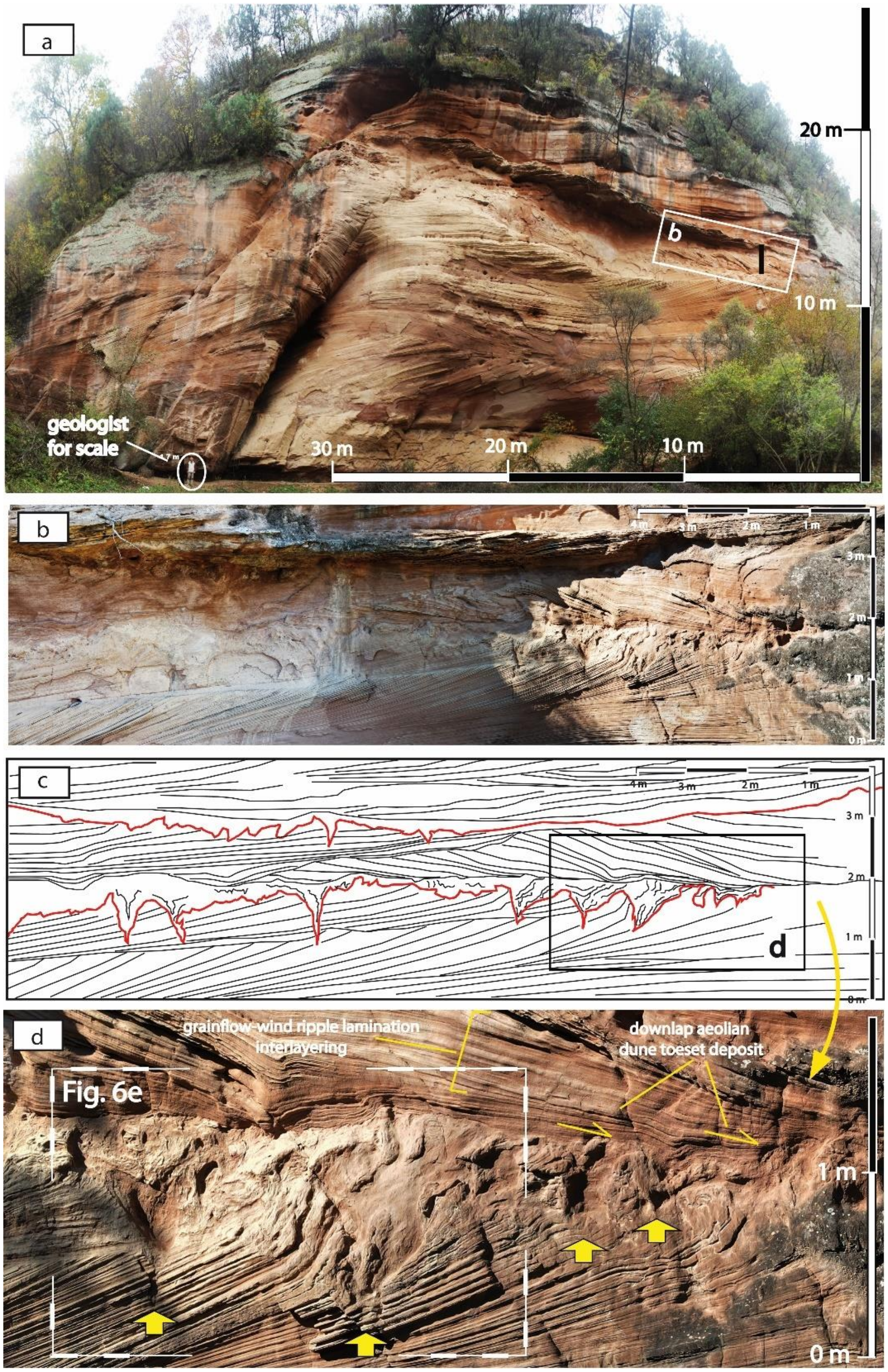

Supplementary Fig. 4 | Aeolian stratigraphic architecture and permafrost wedges. a, Field photograph complex aeolian dune architecture showing location of sandstone wedge horizons in panel (**b**). **b,** Field photograph from (**a**) showing permafrost wedges horizons interbedded with cross-bedded aeolian dune sandstones. **c**, line-drawing of the wedges encased in aeolian dune facies. **d**, Close-up view from (**c**) showing the stratigraphic and sedimentological relationships between the wedges and the host aeolian sandstones. Upward-pointing yellow arrows indicate wedges. Aeolian grain flow deposits sharply overlie the wedge. See detail in Supplementary Fig. 6e.

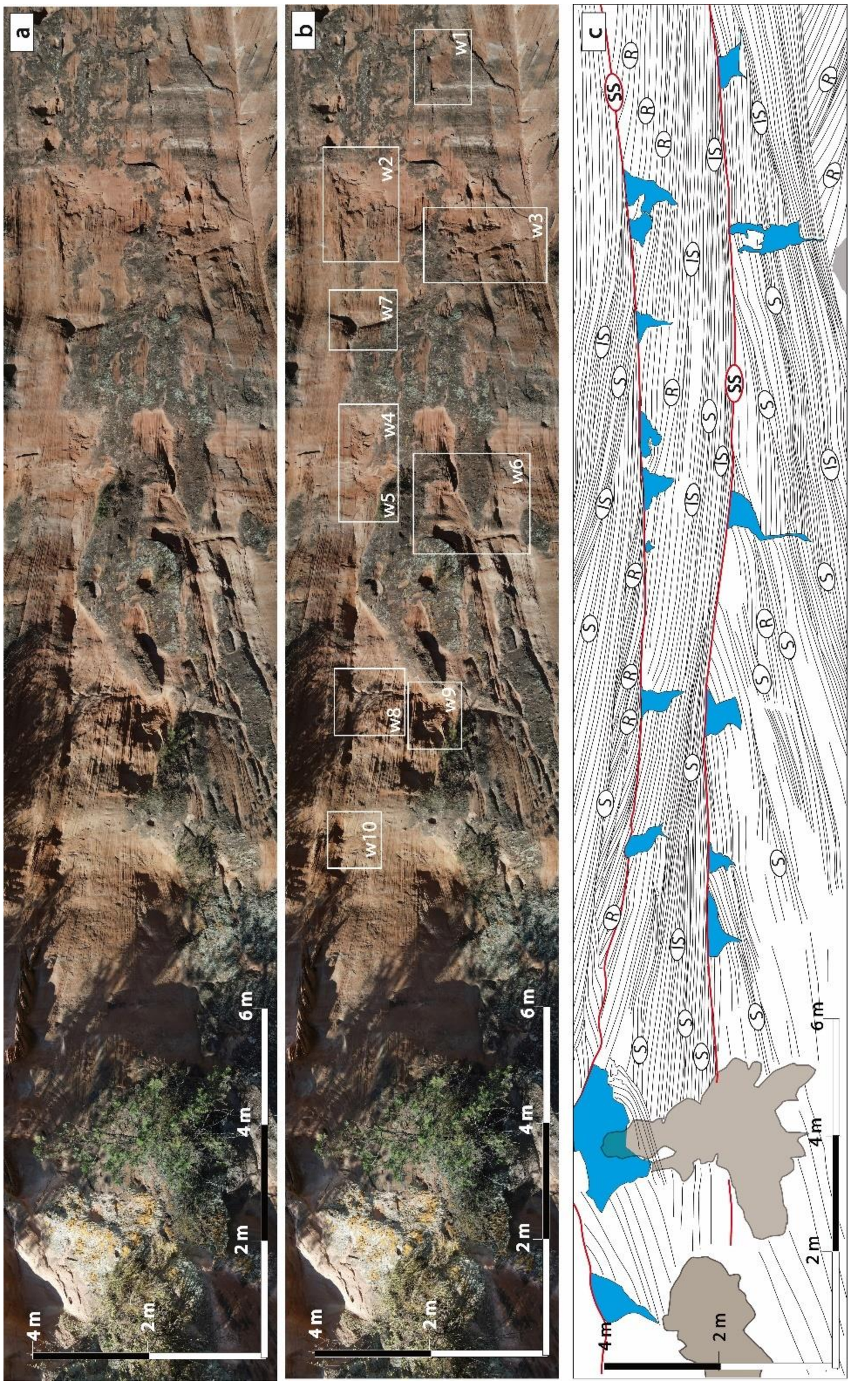

Supplementary Fig. 5 | Cretaceous permafrost wedges and aeolian dune architecture. a, Field photograph of permafrost sandstone wedges horizons hosted in aeolian dune sandstones of the Luohe Fm. **b**, Ten permafrost sandstone wedges are identified (labelled “w1” to “w10”). Detailed sedimentological observations of the wedges can be seen in Fig. 2 and Supplementary Fig. 6. **c**, Aeolian architecture is based on the recognition of aeolian bounding surfaces hierarchy[10,31]. “SS” aeolian supersurface; “IS” interdune surface; “S” superimposition surface; “R” reactivation surface. Permafrost sandstone wedges are marked in blue colour.

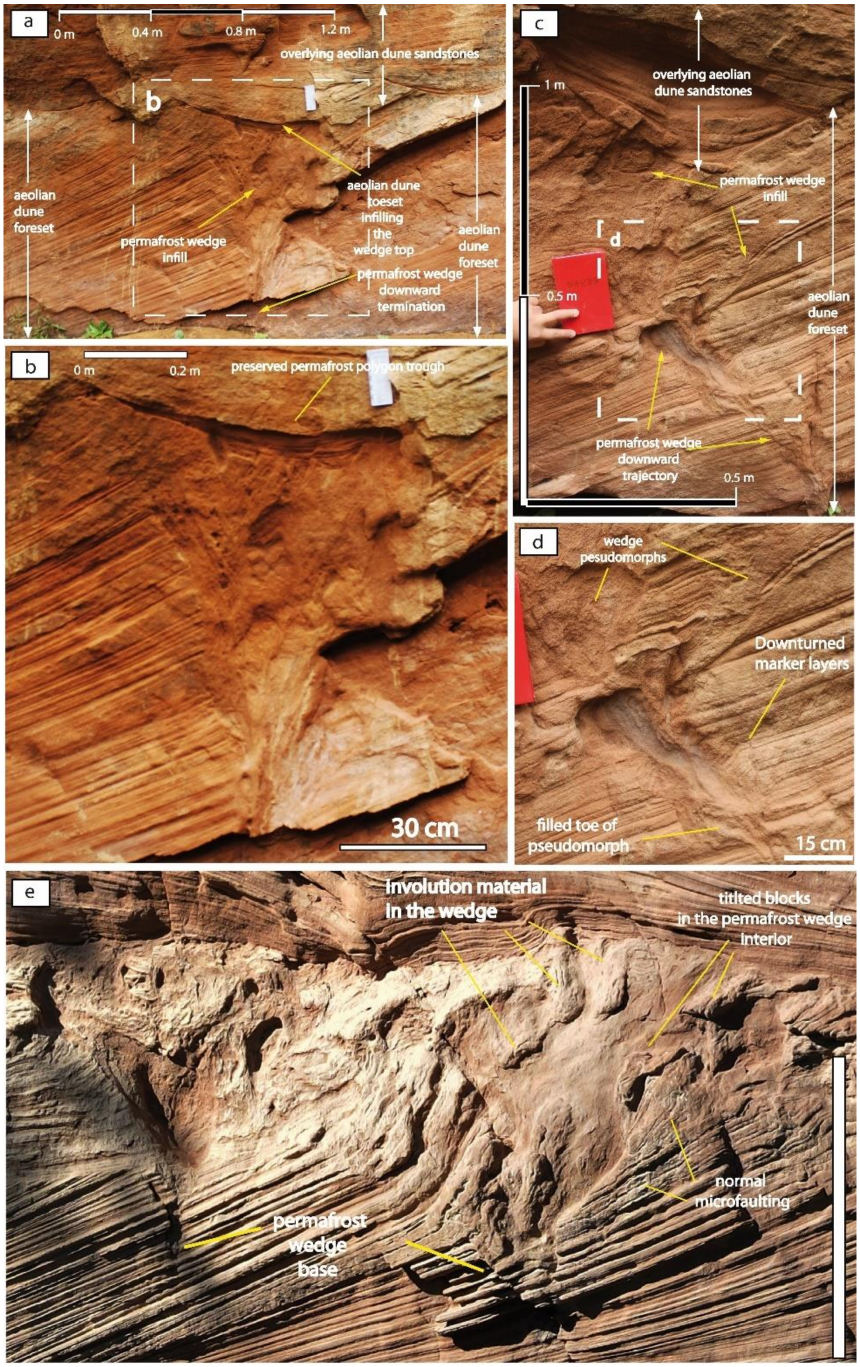

Supplementary Fig. 6 | Detailed sedimentology of the sandstone wedges hosted in aeolian sandstones of the Luohe Fm. a, Permafrost wedge and close-up view (**b**). **c,** Wedge in aeolian dune foreset facies showing infills and downward trajectories covered by aeolian dune sandstones. **d,** Detail from (**c**) showing wedges, and downturned lateral layers of the host aeolian sediments. **e,** Close-up view of two wedges from Supplementary Fig. 4d, showing tilted blocks of host aeolian sands, normal microfaulting and folded pseudomorph tongues penetrating downwards into the cross-bedded aeolian sandstones. Grain flow deposits of the overlying aeolian dune toeset have buried the wedge horizon.

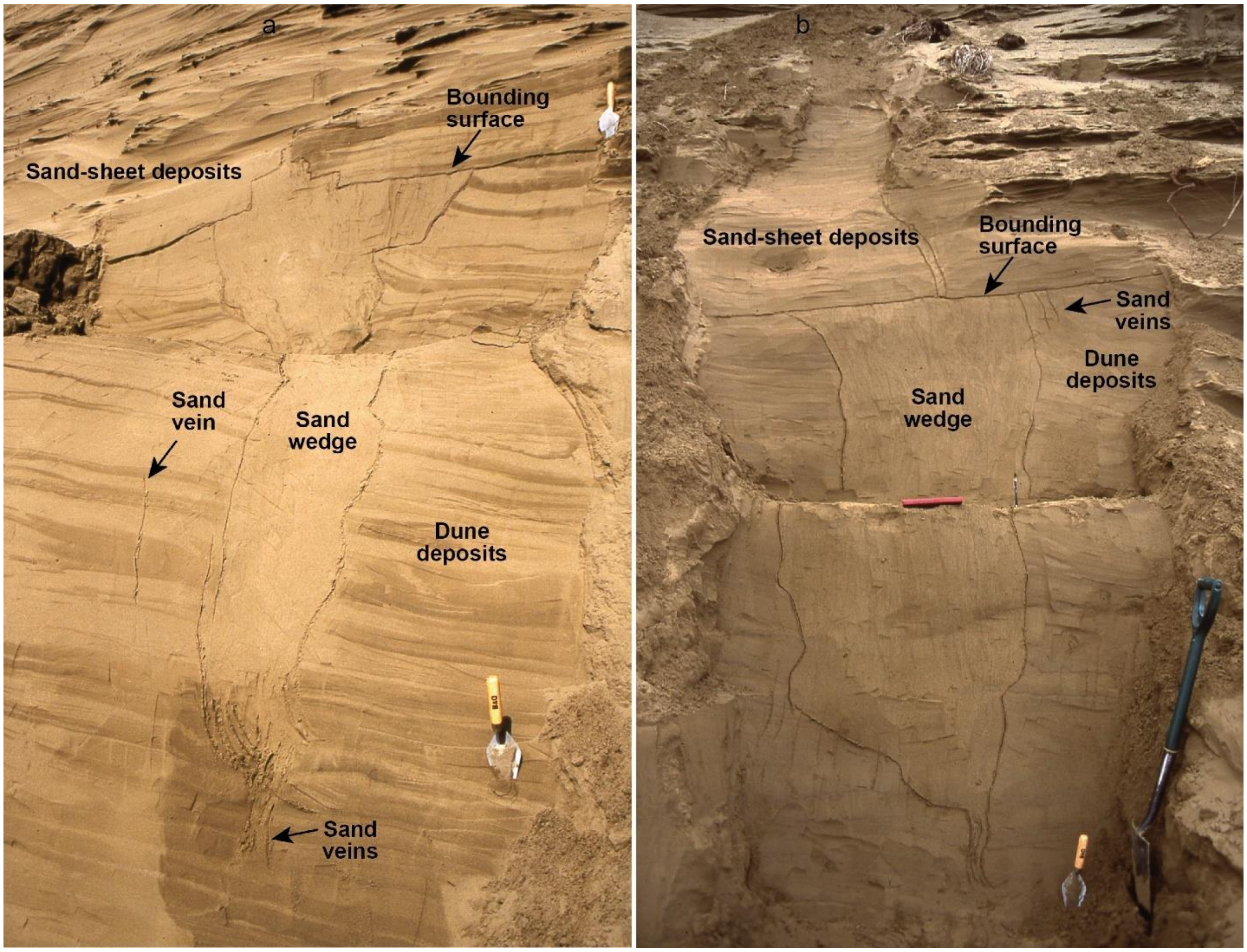

Supplementary Fig. 7 | Sand wedges within aeolian dune deposits and truncated by bounding surfaces, Late Pleistocene Kittigazuit Fm, Hadwen Island, NT, Canada.

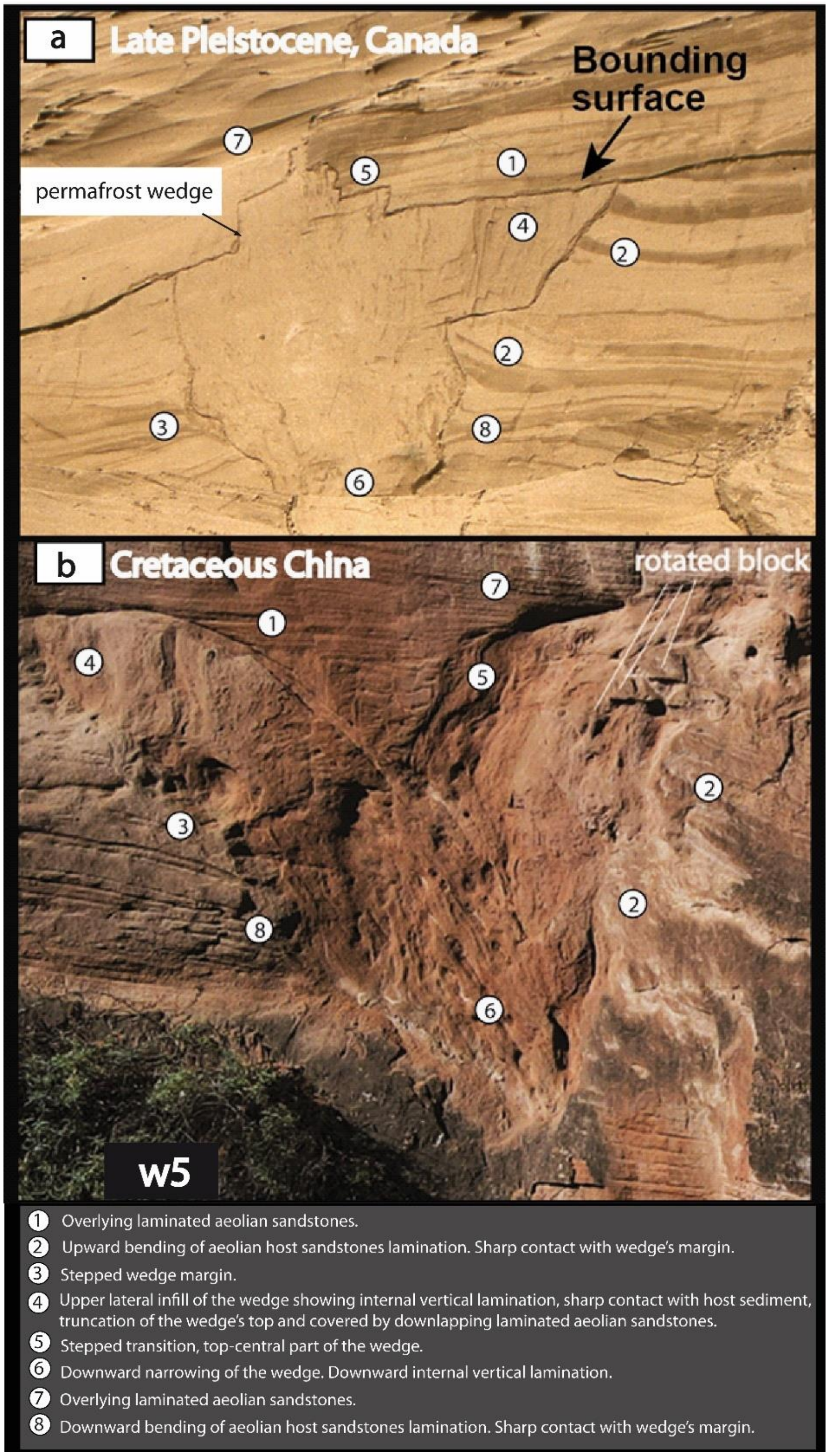

Supplementary Fig. 8 | Late Pleistocene analogue of the Cretaceous permafrost wedges. a, permafrost wedges in aeolian sands of the Late Pleistocene Kittigazuit Fm, Hadwen Island, NT, Canada. Close-up view from Supplementary Fig. 7. **B,** Permafrost wedges in aeolian sandstones of the Cretaceous of China. Numbers 1–8 refer to similar features observed in the permafrost systems of the Pleistocene of Canada (**a**), and the Cretaceous Luohe Fm of China (**b**).

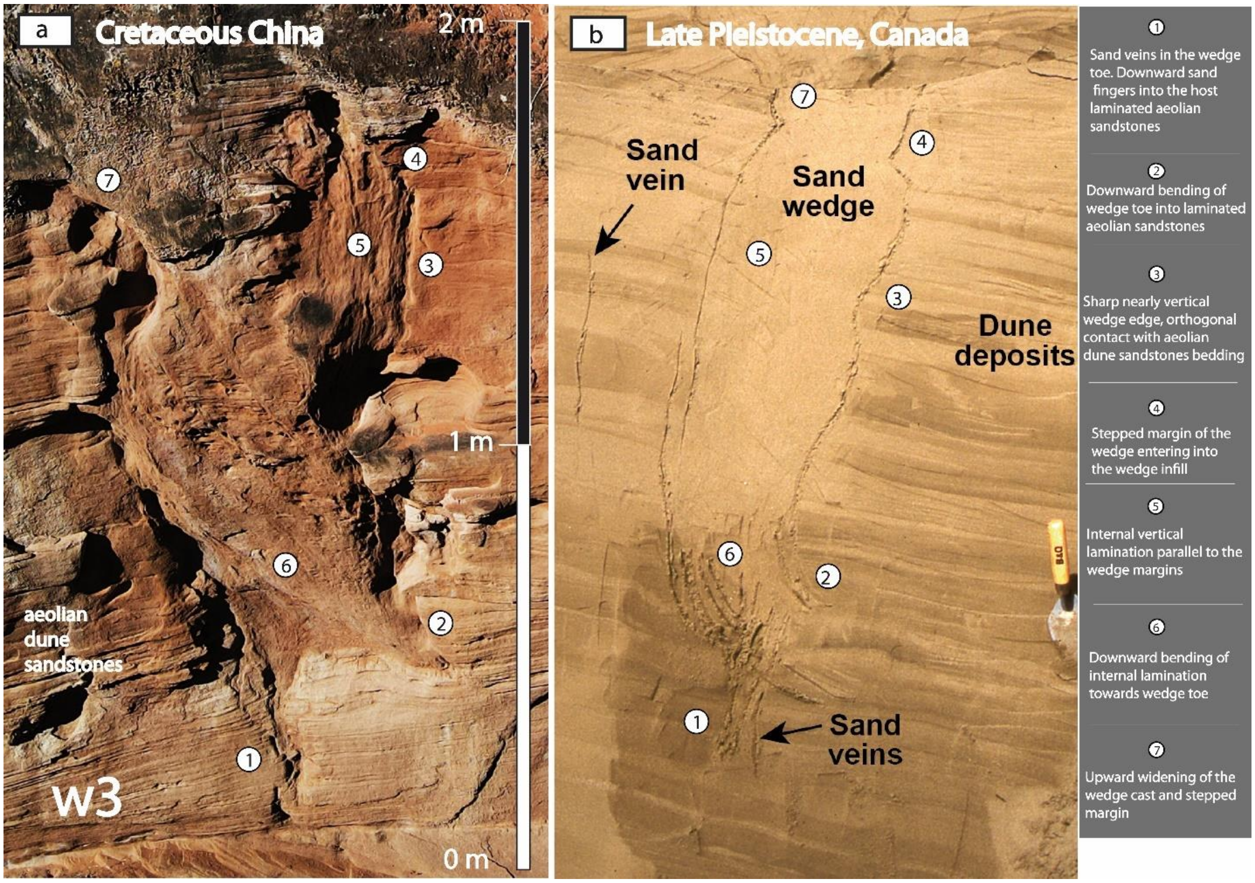

Supplementary Fig. 9 | Late Pleistocene analogue of the Cretaceous permafrost wedges. a, Permafrost wedges in aeolian sands of the Late Pleistocene Kittigazuit Fm, Hadwen Island, NT, Canada. Close-up view from Supplementary Fig. 7. **B,** Permafrost wedges in aeolian sandstones of the Cretaceous Luohe Fm of China. Numbers 1–7 refer to similar features observed in the permafrost systems of the Pleistocene of Canada (**a**), and the Cretaceous of China (**b**).

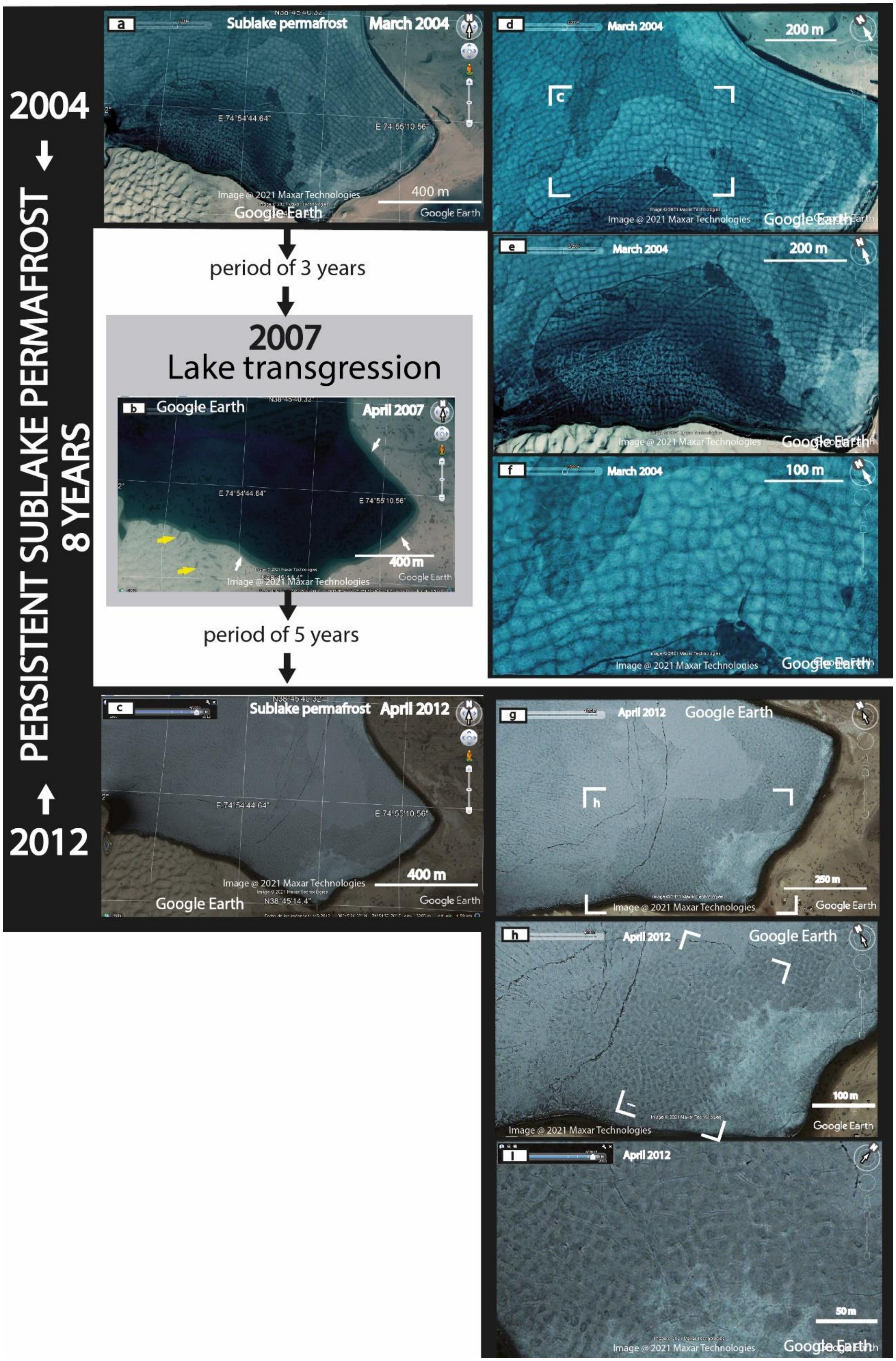
2004
PERSISTENT SUBLAKE PERMAFROST
8 YEARS
2012
a
Sublake permafrost
March 2004
400 m
period of 3 years
2007
Lake transgression
b
Google Earth
April 2007
400 m
period of 5 years
c
Sublake permafrost
April 2012
400 m
d
March 2004
200 m
e
March 2004
200 m
f
March 2004
100 m
g
April 2012
h
April 2012
100 m
i
April 2012
50 m
Image @ 2021 Maxar Technologies
Google Earth

Supplementary Fig. 10 | Evidence of permanent sublake permafrost in the Qiongkuai Lebashi Lake, Xinjiang Uygur Autonomous Region, China. **a**, **b**, and **c**, Satellite images from the same area of the lake. Sublake permafrost thermal contraction crack polygons are visible in both March 2004 and April 2012. The lake suffered a transgression in 2007. Ice floe transparency both in March 2004 (**d**, **e**, **f**) and April 2012 (**g**, **h**, **i**) allowed observations of the sublake permafrost polygons. See Fig. 3e–f for the temporal tracking of individual sublake permafrost polygons. (a-i: Image @2021 Maxar Technologies and Google Earth).

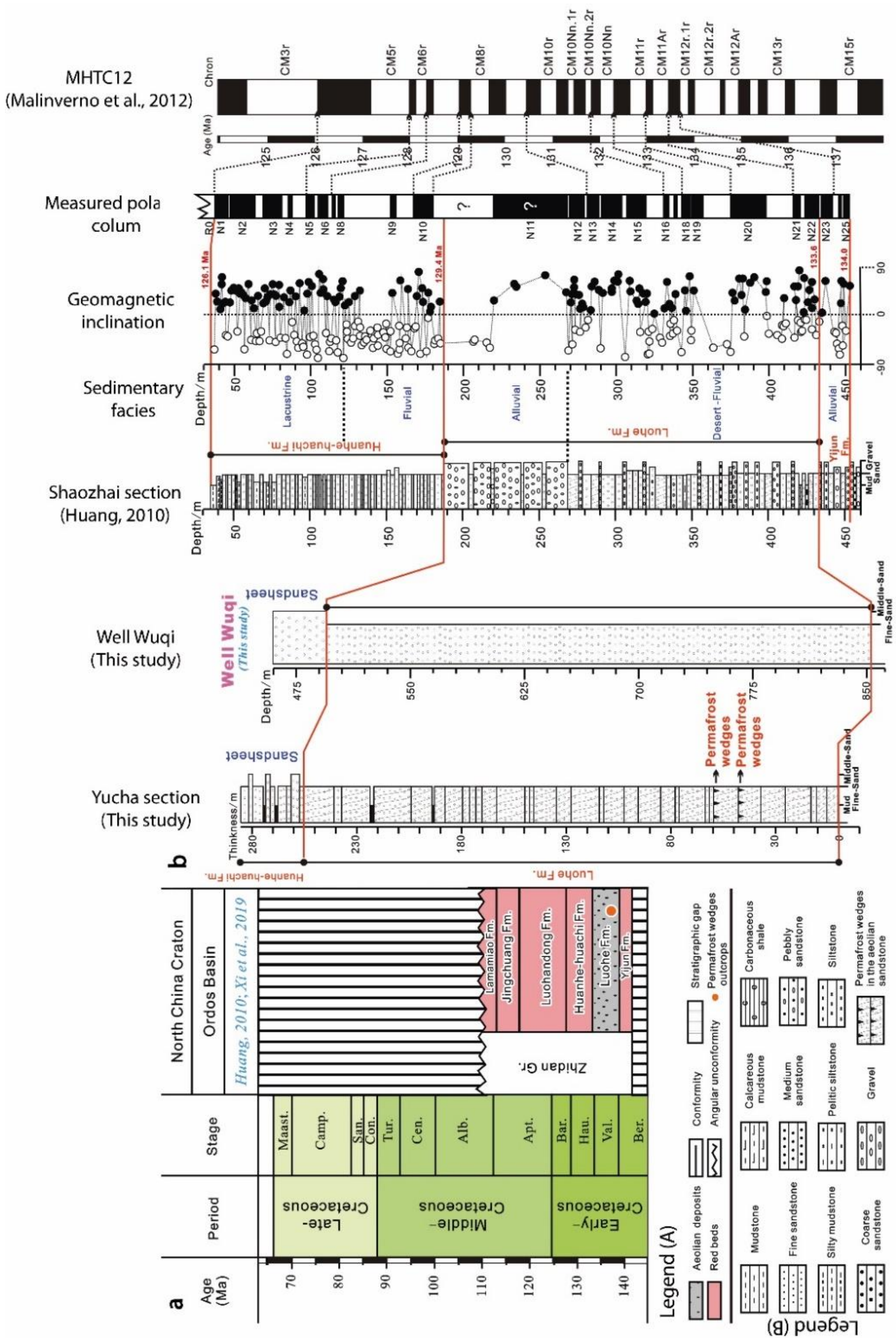

Supplementary Fig. 11| a, Stratigraphic division of Cretaceous in the Ordos Basin[5,63]. **b,** The stratigraphical sections and revised age configuration of the Early Cretaceous Luohe Fm and Huanhe–Huachi Fm in the Ordos Basin. Compared with Yucha (this study), Well Wuqi (this study), and Shaozhai sections[5], combined with the identification of correlation datum for defined by the stratigraphic contact between purplish red medium bedded, medium-grained sandstones, and the underlying red thick-bedded fine-grained sandstones with large high-angle tabular cross-bedding or medium-bedded conglomerates, allowed to obtain an astronomically corrected duration of 133.74–129.4 Ma for the Luohe–Yijun Fm in Well Lingtai, and 133.59–129.4 Ma for the Luohe Fm in Well Wuqi, respectively. The revised age configurations are adjusted by the MHTC12 (ref.[48]).

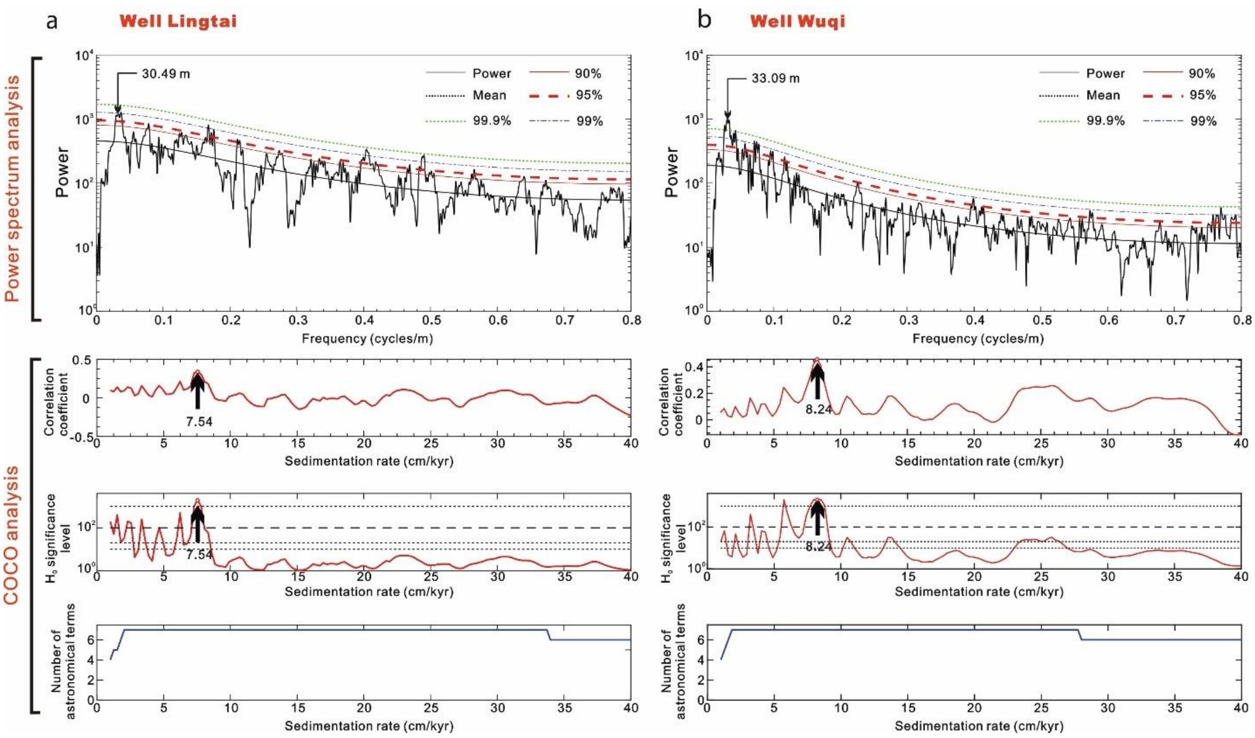

Supplementary Fig. 12| 2π MTM power spectrum and correlation coefficient (COCO) analysis of GR series in Well Lingtai (**a**) and Well Wuqi (**b**). The target series is the La2004 astronomical solution at 132 Ma. Significance levels were estimated using a Monte Carlo simulation with 2,000 iterations.

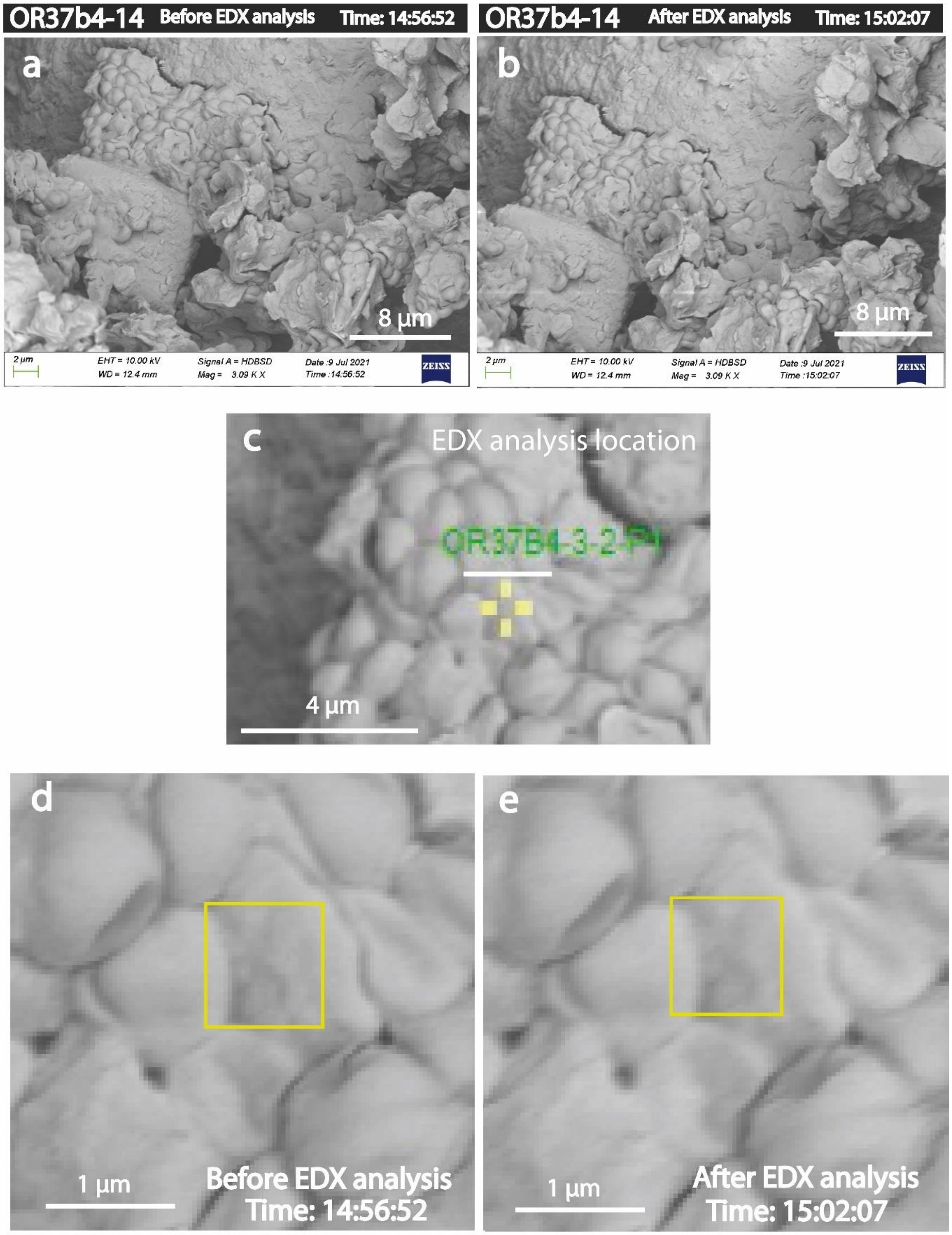

Supplementary Fig. 13 | Sample OR3b4-14. Evidence of fossilization 1. SEM images of bacteria (**a**) before and (**b**) after the electron beam shooting (**c**). A close-up view on the EDX analyzed area before (**d**) and after (**e**). The EDX analysis shows no morphological variation neither dehydration nor shrinkage of cells evidencing a mineralized stage of fossil bacteria.

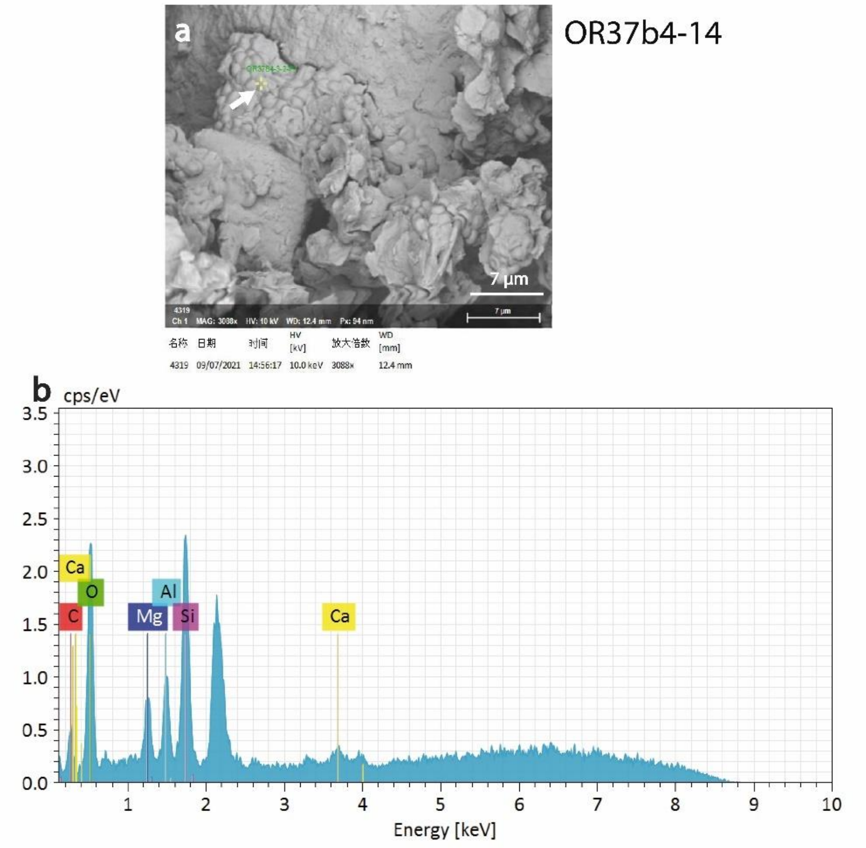

c

Element	Element number	Non-normalized Weight concentration (%)	Normalized Weight concentration (%)	Atomic concentration (%)	Relative error (1σ) (%)
**O**	8	9.01	42.63	48.69	15.59
**Si**	14	5.78	27.36	17.80	4.81
**C**	**6**	**3.31**	**15.67**	**23.84**	**23.24**
**Al**	13	1.44	6.79	4.60	7.12
**Mg**	12	1.16	5.47	4.11	8.35
**Ca**	20	0.44	2.07	0.95	11.50
	**Total:**	**21.13**	**100.00**	**100.00**	

Supplementary Fig. 14 | Sample OR37b4-14. EDX analyses showing evidence of preserved carbon on the fossilized bacterial cells. a, Location of EDX analysis. **b,** Results of EDX analysis. **c,** Major elements present in the analysed point.

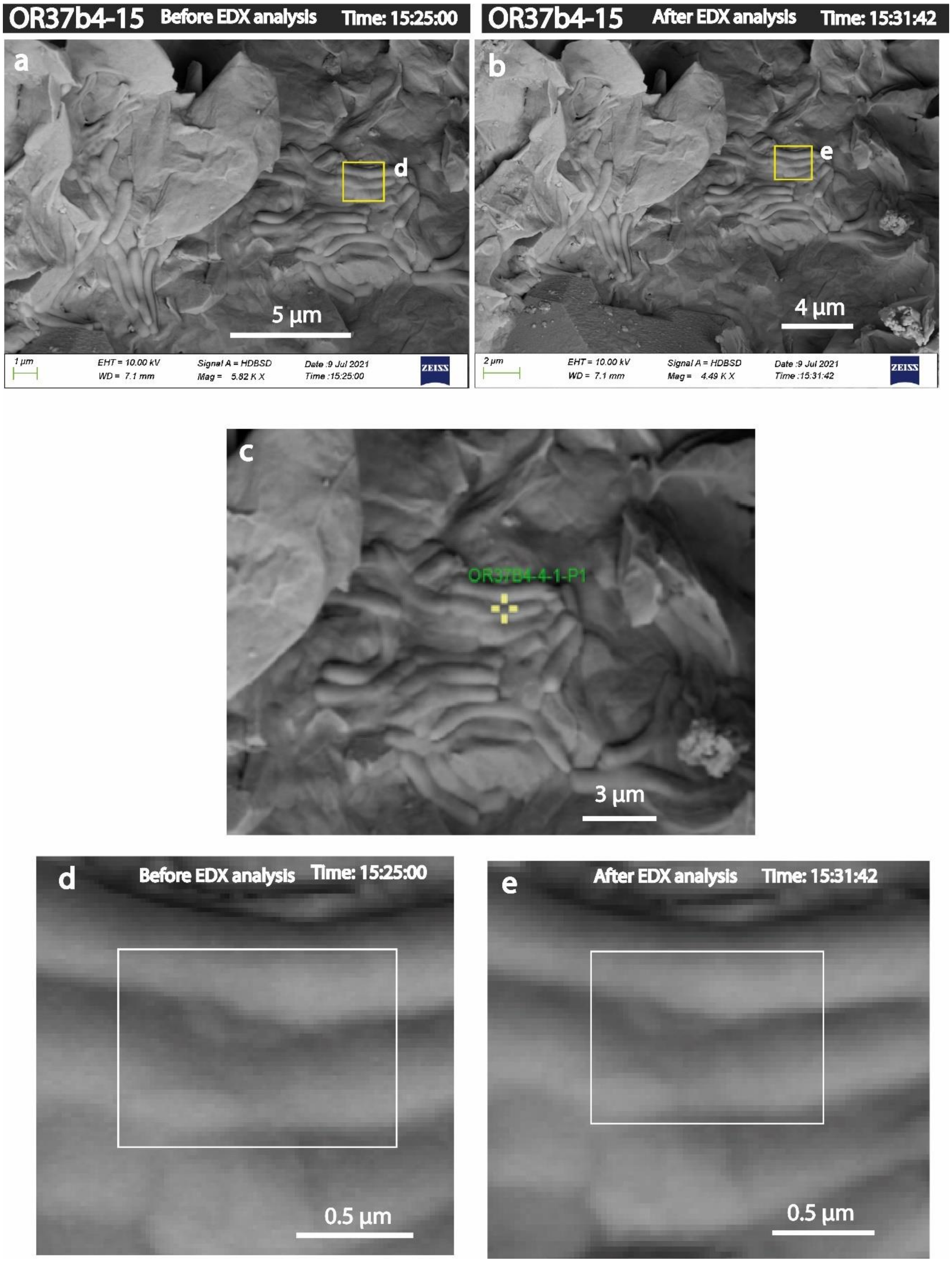

Supplementary Fig. 15 | Sample OR3b4-15. Evidence of fossilization 2. SEM images of bacteria (**a**) before and (**b**) after the electron beam shooting (**c**). A close-up view on the EDX analyzed area before (**d**) and after (**e**) the EDX analysis shows no morphological variation neither dehydration nor shrinkage of cells evidencing a mineralized stage of fossil bacteria.

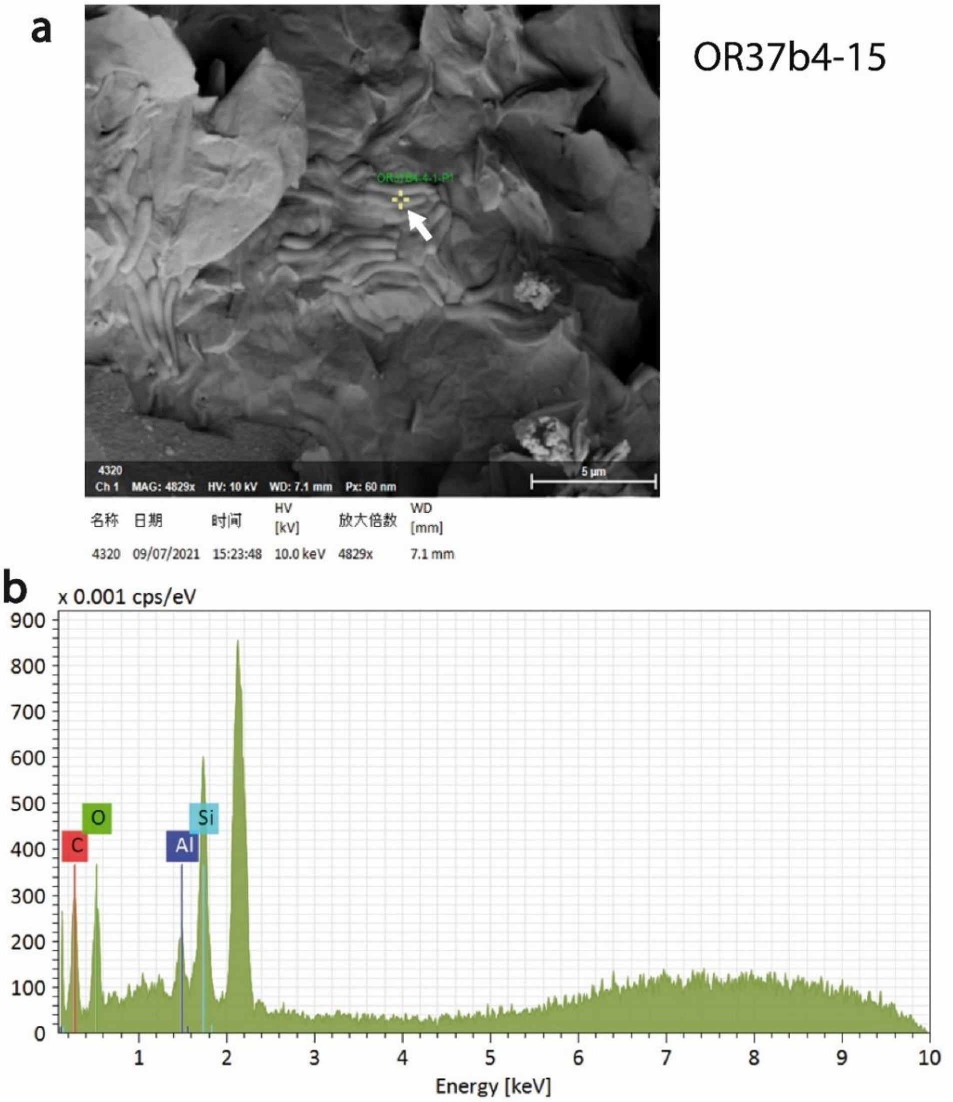

c

Element	Element number	Non-normalized Weight concentration (%)	Normalized Weight concentration (%)	Atomic concentration (%)	Relative error (1σ) (%)
C	6	2.82	43.67	57.59	23.03
**O**	8	1.58	24.55	24.31	24.89
**Si**	14	1.54	23.87	13.46	6.49
**Al**	13	0.51	7.91	4.64	11.46
	**Total:**	**6.45**	**100.00**	**100.00**	

Supplementary Fig. 16 | Sample OR37b4-15. EDX analyses showing evidence of preserved carbon on the fossilized bacterial cells. a, Location of EDX analysis. **b**, Results of EDX analysis. **c**, Major elements present in the analysed point.
