# Supplementary material for: Permafrost in the Cretaceous supergreenhouse
Source: Nat Commun. 2022 Dec 26;13:7946. doi: 10.1038/s41467-022-35676-6 (PMC9792593; doi:10.1038/s41467-022-35676-6)
Supplement: Supplementary file 3 — Description of Additional Supplementary Files [file 41467_2022_35676_MOESM3_ESM.pdf]

Title: Supplementary Data 1:
Description: Well LingtaiLuhoe Fm. (Gamma Ray). Courtesy of Petrochina Changqing Oilfield Company, China.

Title: Supplementary Data 2:
Description: Well Wuqi-Luohe Fm. (Gamma Ray). Courtesy of Petrochina Changqing Oilfield Company, China.
